# Unraveling sphingolipid dynamics in late-onset preeclampsia: insights from lipidomic analysis

**DOI:** 10.11613/BM.2025.010708

**Published:** 2025-02-15

**Authors:** Tamara Antonic, Sandra Vladimirov, Daniela Ardalic, Milica Miljkovic-Trailovic, Marija Saric-Matutinovic, Tamara Gojkovic, Jelena Munjas, Jasmina Ivanisevic, Snezana Jovicic, Jelena Vekic, Aleksandra Zeljkovic, Zeljko Mikovic, Aleksandra Stefanović

**Affiliations:** 1 Department of Medical Biochemistry, Faculty of Pharmacy, University of Belgrade, Belgrade, Serbia; 2 The Obstetrics and Gynecology Clinic “Narodni Front”, Belgrade, Serbia; 3 Department of Obstetrics and Gynecology, Faculty of Medicine, University of Belgrade, Belgrade, Serbia

**Keywords:** high-density cholesterol, late-onset preeclampsia, sphingolipids, sphingosine-1-phosphate

## Abstract

**Introduction:**

Sphingolipids, essential to trophoblast and endothelial function, may impact inflammation in preeclampsia. However, their specific role in late-onset preeclampsia remains unclear. To address this research gap, we analyzed sphingolipid profiles in pregnancies at high risk for preeclampsia development to identify potential biomarkers and clarify their role in disease pathogenesis.

**Materials and methods:**

We monitored 90 pregnant women at high risk for preeclampsia development across four gestational points. These women were later categorized into the group of women with high risk who did not develop preeclampsia (HRG) (70 women) or the preeclampsia group (PG) (20 women). Sphingolipids (sphingosine, sphinganine, sphingosine-1-phosphate (S1P), ceramides C16:0/C24:0, and sphingomyelin C16:0) were quantified via liquid chromatography-tandem mass spectrometry.

**Results:**

Sphingolipid profiles revealed distinct patterns between groups. Concentrations of S1P in the HRG increased from the 1st trimester to delivery (P < 0.001). We did not notice significant changes in S1P during pregnancy in the PG but compared with the HRG we found significantly lower concentrations at each test point from the 2nd trimester until delivery (P = 0.020, P = 0.013, P = 0.011, respectively). Ceramides C16:0 and C24:0 demonstrated significant increases over time in HRG (P < 0.001, both). Sphingomyelin C16:0 increased significantly across pregnancy in both groups (P < 0.001 in HRG and P = 0.006 in PG), with no significant differences between groups.

**Conclusions:**

We identified S1P as a potential biomarker for late-onset preeclampsia, with lower concentrations observed in PG compared to HRG. Rising sphingomyelin concentrations in both cohorts might serve as a relevant cardiovascular risk indicator in pregnancies at high risk for preeclampsia.

## Graphical abstract

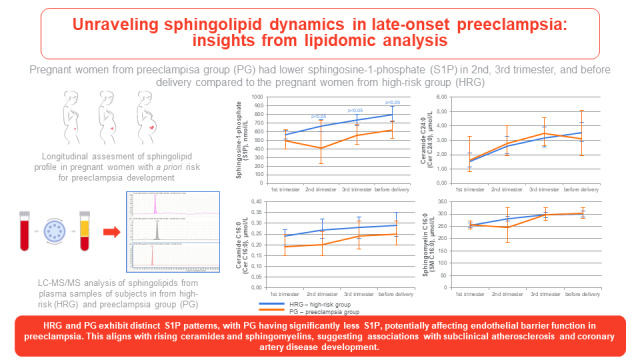

## Highlights

• This study fills gap by exploring sphingolipid profile in late-onset preeclampsia

• Distinct sphingosine-1-phosphate (S1P) patterns were seen in high-risk women without preeclampsia *vs.* preeclampsia group

• Preeclampsia group had lower S1P in 2nd, 3rd trimester, and before delivery

• High-density lipoprotein drop in 2nd trimester in preeclampsia group, aligning with lower S1P concentrations

• Both cohorts showed rising sphingomyelin concentrations

## Introduction

Preeclampsia is a potentially life-threatening pregnancy disorder characterized by high blood pressure (systolic (SBP) / diastolic blood pressure (DBP) exceeding 140/90 mmHg) after the 20th week of gestation in a previously normotensive woman, often accompanied by proteinuria (*1*). It can also occur without significant proteinuria, leading to organ damage (*1*).

Preeclampsia presents in two forms based on the time of onset: early- (developing before the 34th week of gestation) and late-onset (developing after the 34th week of gestation), each with distinct associated risks and potential outcomes, but sharing an incomplete understanding of their underlying causes, believed to involve the intricate interplay between placental dysfunction, immune response, oxidative stress, inflammation, and vascular dysfunction (*2*, *3*). A two-stage model posits initial placental dysfunction, triggering a cascade of molecular mechanisms leading to maternal endothelial dysfunction and multi-organ damage (*3*, *4*). Early-onset preeclampsia is associated with deficient placentation and compromised uteroplacental perfusion, while late-onset preeclampsia may stem from placental compression or syncytiotrophoblast senescence (*2*, *4*, *5*). Also, late-onset preeclampsia is followed by better maternal and perinatal outcomes (*2*, *3*).

Preeclampsia pathogenesis is greatly impacted by disorders of maternal lipid metabolism before and during pregnancy, which is strongly associated with inflammation, oxidative stress, and endothelial dysfunction (*6*). Pregnancy without complications is characterized by elevated total cholesterol, low-density lipoprotein (LDL) cholesterol, and triglycerides alongside increased high-density lipoprotein (HDL) cholesterol (*7*). In preeclampsia, this pattern is often reversed regarding the HDL cholesterol compared to normotensive pregnancies (*8*). Understanding lipid roles in preeclampsia is complex, with ongoing research utilizing mass-spectrometry based lipidomics to identify specific lipid species including sphingolipids (*6*, *9*). These molecules, previously viewed as static, are now recognized as dynamic signaling agents influencing essential cellular functions, with growing attention on their role in preeclampsia pathogenesis (*10*-*12*).

Sphingolipids, particularly ceramides and sphingosine-1-phosphate (S1P), could influence trophoblast functionality, impacting vascular endothelial function and triggering inflammation and oxidative stress in preeclampsia (*10*, *13*). Elevated ceramide concentrations in preeclamptic placentas are thought to instigate augmented autophagic activity of trophoblasts and apoptotic cell death, characteristic hallmarks of this pathological condition (*10*). Stimulation of cell-surface receptors (S1PRs) by S1P leads to cellular proliferation, survival promotion, and increased nitric oxide production, inducing vasorelaxation, and providing atheroprotective effects to the vascular environment (*14*, *15*). Dysfunctional S1P biosynthesis is observed in preeclampsia (*14*).

While sphingolipids have been implicated in preeclampsia, their exact mechanisms and effects remain poorly understood. Most existing studies focus on early-onset preeclampsia, leaving a critical gap in knowledge regarding late-onset cases. Our study addresses this gap by investigating the sphingolipid metabolic profiles, focusing on S1P, ceramides, and sphingomyelins, in pregnant women with high-risk for preeclampsia development, comparing those who developed late-onset preeclampsia to those who remained unaffected. By exploring these metabolic alterations, our research aims to clarify potential role of these sphingolipids as biomarkers for late-onset preeclampsia and to better understand their role in the disease’s pathogenesis.

## Materials and methods

In this retrospective cohort study, 90 pregnant women were systematically monitored across four antenatal assessments at the Obstetrics and Gynecology Clinic “Narodni Front”, Belgrade, Serbia. Ethical clearance was duly obtained from the Ethics Committee of Gynecology and Obstetrics Clinic “Narodni front” (reference No. 05006-2020-10738), the Ethics Commission of the Faculty of Medicine, University of Belgrade (designation No. 1322/VII-27), and the Ethical Committee for Biomedical Research of the Faculty of Pharmacy, University of Belgrade (reference No. 1156/2). Informed written consent was procured from all participants. The study strictly adhered to ethical guidelines outlined in the Declaration of Helsinki, and ensuring compliance with institutional and national regulations for research involving human subjects.

### Subjects

This study forms part of a broader research initiative titled “HIgh-density lipoprotein MetabolOMe research to improve pregnancy outcome - HI-MOM.” The recruitment of study subjects was conducted between October 2016 and March 2018. Pregnant individuals assessed as “at risk for developing pregnancy complications” - based on evaluations at the primary healthcare level - underwent early preeclampsia screening between 11 and 13 weeks of gestation at the Obstetrics and Gynecology Clinic “Narodni Front”, enabling timely intensive monitoring to mitigate pregnancy-related complications. Recruitment followed the National Institute for Health and Care Excellence guidelines, with inclusion criteria based on high- and moderate-risk factors for preeclampsia (*16*). A detailed description of the inclusion and exclusion criteria has been provided in our previous studies (*17*, *18*). The primary outcome monitored in the study was the development of preeclampsia, while also tracking the emergence of secondary outcomes, *i.e.*, other complications, such as hypertensive disorders, intrauterine growth restriction (IUGR), and gestational diabetes.

Initially, there were 114 pregnant women at high risk for preeclampsia development, but 24 were excluded because of withdrawals, miscarriages, or fetal anomalies. Among the 90 women, 20 (22.2%) developed clinical signs of preeclampsia by the end of gestation. Preeclampsia was defined as hypertension (≥ 140/90 mmHg) in women who were normotensive before 20 weeks of gestation, accompanied by proteinuria (≥ 300 mg/24 h) or other signs of systemic organ failure (impaired liver function, renal insufficiency, pulmonary edema, thrombocytopenia, and new-onset cerebral or visual disturbances) (*1*). Among the 70 women (77.8%) not developing preeclampsia, some faced other pregnancy complications. Our previous papers have provided a detailed classification of pregnant women based on their pregnancy complications (*17*, *18*). Notably, 47 women completed pregnancy without complications.

Participants were classified into two groups based on the primary outcome - the group of women with high-risk who did not develop preeclampsia (HRG), comprising individuals without preeclampsia, and the preeclampsia group (PG). The absence of a control group was justified by the fact that women with high risk for preeclampsia development are selected based on established risk factors already implemented in clinical practice; therefore, our focus was on identifying markers that could differentiate those who develop preeclampsia from others within this high-risk population.

Subjects were monitored at four gestational stages: initial (11-14 weeks), second (22-25 weeks), third (28-32 weeks), and before delivery (35-38 weeks). After a 12-hour fast, venous blood samples were collected between 7 a.m. and 9 a.m., using one 10 mL serum tube and one 5 mL EDTA tube (Becton, Dickinson and Company, Franklin Lakes, USA). Samples were centrifuged at 1500xg for 10 minutes to segregate serum or plasma. General lipid profile parameters were analyzed in serum, instantly after sampling, while sphingolipids, apolipoprotein A-I (apoA-I), and apolipoprotein M (apoM) were measured from samples stored at - 80 °C until further analysis (sphingolipids in plasma, apoA-I and apoM in serum).

### Methods

Clinical parameters monitored in the study included uterine artery flow rate (mean pulsatility index), body mass index (BMI), mean arterial pressure (MAP), and weight gain. Pregnant individuals underwent uterine artery flow rate assessment *via* pulse color Doppler throughout the entire pregnancy. The BMI and MAP were calculated using established formulas as detailed in our previous work (*18*, *19*).

Serum lipid parameters, including total cholesterol, HDL cholesterol, LDL cholesterol (calculated using Friedewald’s equation), and apoA-I, were determined using commercially available kits and methods as already explained in details (*17*, *18*). ApoM mass concentrations were quantified using the Enzyme-Linked Immunosorbent Assay (ELISA) technique (FineTest Biotech Inc., Wuhan, China).

High-performance liquid chromatography (HPLC) grade analytical standards were used for quantification of sphingosine (SPH), sphinganine (SAP), S1P, ceramide C16:0 (Cer C16:0), ceramide C24:0 (Cer C24:0) (Avanti Polar Lipids, Birmingham, USA), sphinganine-1-phosphate (SAP1P), and sphingomyelin C16:0 (SM C16:0) (Cayman Chemical, Ann Arbor, USA). SPH d17, S1P d17, and ceramide C17:0 (Cer C17:0), obtained from Avanti Polar Lipids (Birmingham, USA), and sphingomyelin C17:0 (SM C17:0) purchased from Cayman Chemical (Ann Arbor, USA) were used as internal standards (IS). Methanol (HPLC grade) was purchased from Fisher (Pittsburgh, USA), chloroform, and ammonium formate from Sigma Aldrich (St. Louis, USA), trifluoroacetic and formic acid from Thermo Fisher Scientific (Waltham, USA).

Sphingolipid quantification employed liquid chromatography-mass spectrometry/mass spectrometry (LC-MS/MS), beginning with plasma lipid extraction *via* liquid-liquid extraction. A 50 µL plasma aliquot was introduced into the IS-coated (SPH d17, S1P d17, Cer C17:0, and SM C17:0 mixture) glass vials. Subsequently, 2 mL of an extraction mixture (methanol:chloroform blend in a 2:1 ratio with 0.1% trifluoroacetic acid) was added to each vial and vigorously vortexed for 30 seconds. In the ensuing step, 0.67 mL of chloroform was introduced, and the test tubes were once again thoroughly mixed for 30 seconds. Following this, 1.15 mL of HPLC-grade water was added to establish a 1:1:0.9 ratio for methanol, chloroform, and water. After vortexing for 30 seconds and centrifugation for 20 minutes at 2000xg, a protein layer could become distinguishable in the interlayer. The lower chloroform layer was transferred to a new clean tube, dried to solid, and reconstituted in 30 µL of HPLC-grade methanol. It underwent further 30 seconds vortexing and centrifugation at 1500xg for 10 minutes prior to the injection into the column.

Sphingolipids underwent chromatographic separation using a Zorbax Eclipse Plus C8 column (4.6 x 150 mm, 5 µm) (Agilent Technologies, Santa Clara, USA) with a gradient elution of solvent A (1 mM ammonium formate in methanol with 0.2% formic acid) and solvent B (2 mM ammonium formate in water with 0.2% formic acid). The mobile phase initially consisted of 80% A and 20% B, with subsequent linear gradients to 90% A at 10.6 minutes, which was held for 6 minutes. Subsequently, a linear gradient to 99% A was applied up to 66 minutes, and back to 80% A at 68 minutes. The final condition was sustained for further 7 minutes until the end of run. Flow rate was 0.5 mL/min with a 15 µL sample injection volume.

Analyte m/z transitions were provided in Table 1. Quantification was done using multiple-reaction-monitoring (MRM) on triple quad mass spectrometer Agilent 6420 equipped with an electrospray ionization (ESI) ion source (Agilent Technologies, Santa Clara, USA). Source conditions included a gas temperature of 340 °C, vaporizer temperature of 250 °C, gas flow rate of 12 L/min, and nebulizer pressure at 20 psi. Positive and negative capillary voltages were set at 4500 V and 4000 V, respectively.

**Table 1:** Retention times and tandem mass spectrometry conditions for internal standards and selected sphingolipids

**Component**	**Retention time, min**	**MRM transitions (m/z)**	**Collision energy (eV)**
SPH d17 (IS)	6.410	286.3 ® 268.3	56
SPH	7.297	300.3 ® 282.3	4
SAP	7.965	302.3 ® 284.3	8
S1P d17 (IS)	8.132	366.2 ® 250.3	18
S1P	9.265	380.2 ® 264.3	118
SAP1P	9.284	382.2 ® 284.3	125
Cer C24:0	59.977	632.61 ® 264.2	75
Cer C17:0 (IS)	39.261	552.5 ® 264.2	170
Cer C16:0	36.114	520.31 ® 264.2	145
SM C16:0	33.017	703.5 ® 184.0	24
SM C17:0 (IS)	35.933	717.6 ® 184.0	24
SPH d17 - sphingosine d17. SPH - sphingosine. SAP - sphinganine. S1P d17 - sphingosine-1-phosphate d17. S1P - sphingosine-1-phosphate. SAP1P - sphinganine-1-phosphate. Cer C24:0 - ceramide C24:0. Cer C17:0 - ceramide C17:0. Cer C16:0 - ceramide C16:0. SM C16:0 - sphingomyelin C16:0. SM C17:0 - sphingomyelin C17:0. IS - internal standard. MRM - multiple reaction monitoring.			

Intra-run variabilities ranged from 3.8% to 18.8%. Inter-run variabilities varied from 4.1% to 19.4%. Calibration ranges were: SPH (3.91-1001.7 nmol/L), SAP (3.50-223.7 nmol/L), S1P (10.3-1317.6 nmol/L), SAP1P (1.02-524.2 nmol/L), Cer C16:0 (0.055-14.1 µmol/L), Cer C24:0 (0.083-17.8 µmol/L), and SM C16:0 (7.89-1010.0 µmol/L).

### Statistical analysis

Continuous variables were expressed as median and interquartile range (IQR). Friedman Test was applied to test the difference between the data in the function of time. If Friedman Test was significant, Wilcoxon Signed Ranks Test was performed to examine at which time point differences actually occur. Bonferroni adjustment was applied on the results obtained from the Wilcoxon Signed Ranks Test because of multiple comparisons (P adjusted = 0.05/6). The Mann-Whitney U Test was utilized to compare medians between the two study groups. Computed achieved study power exceeded 0.8.

All statistical tests were considered statistically significant at the 0.05 probability level. Statistical analyses were performed with the PASW Statistics 18 (IBM, Armonk, USA), and G*Power 3.1.9.7 (developed by Faul F, Erdfelder E, Lang AG, and Buchner A) (*20*).

## Results

All study participants were Caucasian. The median age was 32 years (IQR: 28 - 37) in the HRG and 35 years (IQR: 30 - 36) in the PG, with no statistically significant difference observed between the groups (P = 0.299).

Table 2 summarizes clinical and lipid profile parameters monitored across follow-up visits in both cohorts. The present analysis reveals that the PG cohort exhibited significantly higher MAP during whole course of pregnancy (P = 0.018 in the 1st trimester, P = 0.005 in the 2nd trimester, P = 0.001 in the 3rd trimester, and P < 0.001 before delivery). BMI was significantly elevated in the PG even preceding gestation (median: 22.8 kg/m^2^; IQR: 20.5 - 26.6 kg/m^2^ in the HRG *vs.* median: 25.9 kg/m^2^; IQR: 23.0 - 29.9 kg/m^2^ in the PG, P = 0.014), and the difference persisted in the 1st trimester (P = 0.008) (Table 2).

**Table 2:** Changes in basic clinical and lipid profile parameters in the group of women with high-risk who did not develop preeclampsia and the preeclampsia group

	**1st trimester**		**2nd trimester**		**3rd trimester**		**37th WG** **(before delivery)**			
	**HRG** **(N = 70)**	**PG** **(N = 20)**	**HRG** **(N = 70)**	**PG** **(N = 20)**	**HRG** **(N = 70)**	**PG** **(N = 20)**	**HRG** **(N = 70)**	**PG** **(N = 20)**	**P_1_**	**P_2_**
WG	12.7 (12.1 - 13.3)	12.6 (12.3 - 13.2)	23.4 (22.7 - 23.7)	23.6 (22.6 - 24.0)	29.6 (28.4 - 30.7)	29.7 (28.6 - 30.9)	36.7 (36.3 - 37.6)	36.3 (35.7 - 37.5)		
MAP, mmHg	85.0 (77.0 - 94.5)	92.0^‖**^ (85.5 - 99.3)	82.2^†¶^ (75.8 - 89.2)	91.3^‖**^ (83.6 - 97.6)	82.5 (78.2 - 91.8)	93.6^‖**^ (86.5 - 100.9)	88.7^‡¶,§¶^ (80.8 - 94.2)	96.7^‡¶,‖¶^ (91.7 - 105.6)	< 0.001	0.007
BMI, kg/m^2^	23.6 (21.1 - 27.5)	27.5^‖**^ (24.2 - 31.1)								
Weight gain, kg	2.00 (1.20 - 4.10)	3.00 (0.25 - 4.40)	5.60^†¶^ (3.70 - 7.10)	4.70 (2.82 - 6.75)	3.20^†**,‡¶^ (2.30 - 4.70)	3.75 (1.60 - 5.17)	3.80^†**,‡¶^ (2.00 - 5.00)	4.30 (1.25 - 6.00)	< 0.001	0.036
Weight gain, %	4.00 (2.00 - 6.00)	4.00 (2.25 - 5.90)	7.00^†¶^ (5.00 - 10.0)	5.50 (4.00 - 8.75)	5.00^‡¶^ (3.00 - 6.00)	5.00 (2.00 - 6.75)	5.00^‡¶^ (3.00 - 6.00)	4.65 (1.25 - 7.00)	< 0.001	0.109
TC, mmol/l	5.19 (4.71 - 6.17)	5.31 (5.11 - 5.85)	6.88^†¶^ (5.85 - 7.56)	6.47^†¶^ (5.54 - 7.42)	7.29^†¶,‡¶^ (6.06 - 8.57)	6.75^†¶^ (5.98 - 8.09)	7.21^†¶,‡¶^ (6.21 - 8.90)	7.41^†¶,‡**^ (5.95 - 8.17)	< 0.001	< 0.001
HDL-C, mmol/l	1.77 (1.53 - 2.01)	1.75 (1.45 - 1.97)	2.10^†¶^ (1.85 - 2.45)	1.83^‖**^ (1.66 - 2.10)	2.00^†¶,‡**^ (1.74 - 2.35)	1.85 (1.70 - 2.12)	1.90^†¶,‡**^ (1.71 - 2.26)	1.84 (1.73 - 2.16)	< 0.001	0.108
LDL-C, mmol/l	2.89 (2.33 - 3.45)	2.71 (2.26 - 3.34)	3.88^†¶^ (3.02 - 4.65)	3.19^†¶^ (2.90 - 4.38)	4.07^†¶,‡¶^ (3.17 - 5.22)	3.14^†**^ (2.61 - 4.88)	3.89^†¶^ (3.19 - 5.11)	3.08 (2.28 - 4.84)	< 0.001	0.012
ApoA-I, g/L	1.93 (1.66 - 2.20)	1.84 (1.65 - 2.39)	2.31^†¶^ (2.06 - 2.58)	2.27^†¶^ (1.95 - 2.68)	2.31^†¶^ (1.97 - 2.54)	2.26^†¶^ (2.01 - 2.53)	2.24^†¶^ (1.95 - 2.46)	2.30^†**^ (2.02 - 2.67)	< 0.001	< 0.001
ApoM, mg/L	54.6 (40.7 - 66.0)	37.7^‖**^ (19.0 - 54.1)	33.0^†¶^ (26.0 - 41.7)	40.3^‖**^ (26.2 - 63.1)	24.4^†¶,‡¶^ (15.9 - 31.7)	18.2^†**,‡¶,‖**^ (10.4 - 22.7)	37.0^†**,§¶^ (21.3 - 54.1)	35.3 (11.0 - 40.7)	< 0.001	0.014
Wilcoxon Signed Ranks Test: ^†^significantly different from the 1^st^ trimester. ^‡^significantly different from the 2^nd^ trimester. ^§^significantly different from the 3^rd^ trimester. Mann-Whitney U Test: ^‖^significantly different from the HRG. ^¶^P < 0.001.**P < 0.05. Data are shown as median (interquartile range, IQR). HRG - the group of women with high-risk who did not develop preeclampsia. PG - preeclampsia group. WG - week of gestation. MAP - mean arterial pressure. TC - total cholesterol. HDL-C - high density lipoprotein cholesterol. LDL-C - low density lipoprotein cholesterol. ApoA-I - apolipoprotein A-I. ApoM - apolipoprotein M. P_1_ - Friedman Test for HRG. P_2_ - Friedman Test for PG. P < 0.05 was considered statistically significant.										

Both groups exhibited a significant rise in total and LDL cholesterol concentrations during the 2nd trimester, with no intergroup differences. HDL cholesterol increased in the HRG but not in the PG, where it was significantly lower compared to the HRG (P = 0.020). An increase in apoA-I mass concentration was observed in the 2nd trimester in both groups (Table 2). A significant decline in apoM concentrations was seen during the 2nd trimester in the HRG and the 3rd trimester in the PG. Compared to the HRG, apoM concentrations in the PG were significantly lower in the 1st trimester (P = 0.008) and 3rd trimester (P = 0.043) but significantly higher in the 2nd trimester (P = 0.007).

Sphingolipid concentrations showed distinct patterns between the examined cohorts (Table 3). SPH and SAP concentrations increased in both groups throughout pregnancy, but increase in SPH concentrations was statistically significant only in HRG. Notably, S1P concentrations in the HRG increased from the 1st trimester to delivery, while there was no significant changes in S1P during pregnancy in the PG. However, S1P concentrations in the PG remained lower than the HRG at all time points, with significant differences in the 2nd (P = 0.020) and 3rd trimesters (P = 0.013), as well as before delivery (P = 0.011).

**Table 3:** Changes in sphingolipids concentrations in the group of women with high-risk who did not develop preeclampsia and the preeclampsia group

	**1*s*t trimester**		**2nd trimester**		**3rd trimester**		**37th WG** **(before delivery)**			
	**HRG** **(N = 70)**	**PG** **(N = 20)**	**HRG** **(N = 70)**	**PG** **(N = 20)**	**HRG** **(N = 70)**	**PG** **(N = 20)**	**HRG** **(N = 70)**	**PG** **(N = 20)**	**P_1_**	**P_2_**
WG	12.7 (12.1 - 13.3)	12.6 (12.3 - 13.2)	23.4 (22.7 - 23.7)	23.6 (22.6 - 24.0)	29.6 (28.4 - 30.7)	29.7 (28.6 - 30.9)	36.7 (36.3 - 37.6)	36.3 (35.7 - 37.5)		
SPH, nmol/l	63.2 (52.7 - 78.2)	71.7 (62.0 - 94.9)	74.2 (60.0 - 89.9)	80.5 (63.2 - 99.9)	80.4^†**^ (61.8 - 101.8)	93.7 (76.6 - 110.9)	101.6^†¶,‡**^ (71.7 - 117.9)	105.6^‡**^ (76.5 - 141.4)	< 0.001	0.064
SAP, nmol/l	34.3 (19.2 - 42.8)	43.0^‖**^ (35.1 - 66.1)	43.4^†¶^ (30.9 - 62.2)	50.9 (33.5 - 64.2)	56.2^†¶,‡¶^ (38.2 - 74.8)	57.5 (43.6 - 68.4)	65.7^†¶,‡¶,§**^ (46.8 - 87.5)	84.3^†**,‡**,§**^ (58.1 – 103.5)	< 0.001	< 0.001
S1P, nmol/l	532.9 (452.3 - 736.5)	498.3 (376.1 - 700.1)	618.9^†¶^ (487.2 - 950.3)	565.3^‖**^ (393.3 - 698.0)	748.6^†¶^ (567.5 - 986.3)	597.4^‖**^ (385.2 - 826.7)	829.9^†¶,‡**,§¶^ (609.6 - 1087.9)	611.6^‖**^ (519.8 - 731.6)	< 0.001	0.791
SAP1P, nmol/l	121.6 (89.3 - 150.6)	110.6 (82.3 - 142.7)	124.2 (90.3 - 170.4)	109.6 (94.6 - 127.0)	131.7 (94.7 - 169.7)	99.2 (83.5 - 142.3)	116.8 (96.7 - 159.3)	124.2 (88.9 - 152.4)	0.917	0.776
Cer C16:0, µmol/L	0.27 (0.15 - 0.39)	0.16 (0.14 - 0.32)	0.32^†¶^ (0.18 - 0.45)	0.21^‖**^ (0.13 - 0.33)	0.31^†¶^ (0.20 - 0.49)	0.24 (0.16 - 0.35)	0.31^†¶^ (0.20 - 0.47)	0.26 (0.18 - 0.33)	< 0.001	0.004
Cer C24:0, µmol/L	1.94 (1.11 - 3.65)	2.48 (1.71 - 4.50)	4.10^†¶^ (1.86 - 6.39)	3.13 (1.32 - 3.56)	4.57^†¶^ (2.77 - 6.54)	3.74 (2.66 - 6.76)	5.08^†¶,‡**^ (3.43 - 6.90)	3.26 (2.41 - 7.39)	< 0.001	0.356
SM C16:0, µmol/L	246.4 (228.1 - 281.3)	254.2 (234.8 - 269.7)	275.2^†¶^ (253.0 - 303.3)	274.8 (240.3 - 300.1)	294.6^†¶,‡¶^ (267.9 - 318.5)	289.7^†**^ (266.3 - 325.4)	294.0^†¶,‡¶^ (272.1 - 320.8)	295.8^†¶,‡**^ (270.6 - 334.4)	< 0.001	< 0.001
Wilcoxon Signed Ranks Test: ^†^significantly different from the 1st trimester. ^‡^significantly different from the 2nd trimester. ^§^significantly different from the 3rd trimester. Mann-Whitney U Test: ^‖^significantly different from the HRG. *P < 0.001; **P < 0.05. Data are shown as median (interquartile range, IQR). HRG - the group of women with high-risk who did not develop preeclampsia. PG - preeclampsia group. WG - week of gestation. SPH - sphingosine. SAP - sphinganine. S1P - sphingosine-1-phosphate. SAP1P - sphinganine-1-phosphate. Cer C16:0 - ceramide C16:0. Cer C24:0 - ceramide C24:0. SM C16:0 - sphingomyelin C16:0. P_1_ - Friedman Test for HRG. P_2_ - Friedman Test for PG. P < 0.05 was considered statistically significant.										

Ceramide concentrations demonstrated significant increases over time in HRG. Although some increase has been noted in PG throughout the pregnancy, the concentrations were significantly lower compared to the HRG in the 2^nd^ trimester for Cer C16:0. SM C16:0 concentrations increased significantly across pregnancy in both groups, with no significant differences observed between the HRG and PG (Table 3).

## Discussion

This study addresses a gap by examining late-onset preeclampsia’s metabolic profiles, focusing on S1P, ceramides, and sphingomyelins in pregnant women who despite high risk did not develop preeclampsia and those with the condition. Our study results pointed out S1P as a potential biomarker for late-onset preeclampsia, with significantly lower concentrations observed in PG. Additionally, the increased concentrations of SM C16:0 in both cohorts highlight its potential as a cardiovascular risk indicator in pregnancies with high risk for preeclampsia development.

Unconventional lipid metabolism is a recognized feature of preeclampsia pathogenesis. In the previously published papers, we investigated the role of cholesterol in late-onset preeclampsia using the same cohort of pregnant women, where general lipid profile parameters were analyzed and reported (*17*, *18*). Similar to our observations, studies report higher triglycerides, lower HDL, and higher LDL cholesterol in preeclamptic pregnancies compared to uncomplicated ones (*7*, *8*). However, these alterations might not universally apply to every case of preeclampsia, and their significance remains unclear. Understanding these changes may reveal links between oxidative stress, inflammation, and endothelial dysfunction, offering insights into preeclampsia pathophysiology and potential therapeutic interventions (*6*).

Building on prior work, this study analyzed longitudinal sphingolipid profiles in pregnant women who were at high risk for developing preeclampsia, comparing pregnant women at risk who did not develop preeclampsia to those with late-onset preeclampsia to identify novel predictive biomarkers and clarify its metabolic underpinnings (*17*, *18*).

Previous studies have reported inconsistent findings regarding sphingolipids concentrations, particularly SPH and S1P, in preeclampsia (*21*, *22*). Some observed no differences in plasma SPH between uncomplicated pregnancies and those complicated by preterm or term preeclampsia at either 14+0 to 17+6 weeks or 18+0 to 24+6 weeks, even when longitudinal measurements from the same individuals were included (*21*). Others reported significantly higher SPH in preeclamptic pregnancies (*22*). Our findings align more closely with those of Johnstone *et al.*, as we observed no significant differences in SPH concentrations between HRG and PG (21). However, a significant increase in SPH was noted in the HRG during the 3rd trimester (28-32 weeks of gestation), suggesting that significant alterations may emerge later in pregnancy. Although the PG showed a nonsignificant trend of rising SPH concentrations, possibly due to small sample size, SPH’s potential role in anti-proliferative processes underscores the need for further research to clarify its importance in preeclampsia pathogenesis, particularly in the later stages of pregnancy (*22*).

The trend in S1P concentrations changes emerged as particularly captivating. S1P concentrations in HRG displayed a consistent incremental trend over the course of pregnancy. In contrast, S1P concentrations in the PG remained consistently lower compared to the HRG during the 2nd and 3rd trimesters, as well as before delivery (Table 3).

Our findings contrast with prior research on S1P in uncomplicated pregnancies and preeclampsia. Past research suggests S1P remains stable in pregnancies without complications, and that S1P concentrations in preeclampsia across trimesters resemble those in pregnancies without complications (*12*, *21*). Despite this, another research found increased S1P in maternal plasma in preeclampsia compared to the healthy pregnant women (*22*). Recent findings indicate decreased S1P bound to HDL in preeclampsia, highlighting its complex role in the condition (*23*). HDL-bound S1P reinforces the endothelial barrier more effectively than albumin-bound S1P (*24*). In our study, PG showed a significant reduction in HDL cholesterol and lower S1P concentrations during the 2nd trimester (Tables 2 and 3). This decrease in S1P may be linked to reduced HDL-bound S1P, contributing to endothelial dysfunction. Moreover, apoM, a specific carrier of S1P, regulates HDL-S1P homeostasis by facilitating S1P release from erythrocytes, protecting it from degradation and enhancing HDL-S1P concentrations (*25*). We observed dynamic apoM patterns, with significant differences between HRG and PG (Table 2). Notably, while apoM concentrations were lower at the beginning and end of pregnancy in PG, they were higher in the 2^nd^ trimester, coinciding with significantly lower S1P and HDL cholesterol. This phenomenon might bear protective implications for the PG, considering that apoM potentially enhances the anti-atherosclerotic functionalities of the HDL-S1P complex. However, other HDL components may also affect S1P concentrations. The lipophilic pocket of apoM, designed for S1P conveyance, may also interact with other molecules, suggesting alternative partners for S1P within HDL (*26*, *27*). This underscores the complexity of S1P regulation and the need for further research to elucidate its precise role in preeclampsia pathogenesis.

Prior research indicates elevated Cer C16:0, Cer C18:0, and Cer C24:0 during pregnancy without complications, suggesting the involvement of pro-inflammatory ceramides in parturition, similar to pro-inflammatory cytokines (*12*, *22*). Alternatively, increased pro-apoptotic ceramides during late pregnancy may be released from placental syncytial knots as part of the placental trophoblast turnover (*28*). Our study showed increasing ceramide concentrations, though this pattern was observed exclusively in the HRG. Certain ceramide species, including Cer C14:0 and Cer C24:0, are proposed to be elevated in preeclampsia, correlating with severe symptoms (*29*). However, we observed significantly lower Cer C16:0 in the PG during the 2nd trimester, without other significant differences in ceramide concentrations between the groups.

A previous study found rising plasma sphingomyelin concentrations in both preeclamptic and control groups across gestation, with higher SM C18:0 concentrations in late gestation in preeclamptic patients (*12*, *30*). Likewise, we found increasing SM C16:0 concentrations throughout pregnancy in both groups, but no significant differences between the HRG and PG, possibly due to the exclusion of pregnant women without preeclampsia risk factors. Elevated plasma sphingomyelin concentrations are extensively linked to subclinical atherosclerosis and coronary artery disease, suggesting their potential as indicators of subsequent cardiovascular complications in women with a history of preeclampsia (*12*).

One notable limitation of the study is the relatively small PG sample size, which may influence the robustness of the findings. To overcome this limitation, it is crucial to increase participant numbers in future studies.

In summary, our findings emphasize the potential of S1P as a key biomarker in late-onset preeclampsia, distinguishing it from other sphingolipids that showed no significant differences between cohorts. The observed significantly lower S1P concentrations in the PG suggest its importance in understanding preeclampsia pathogenesis and its potential utility for early detection and targeted interventions. Rising sphingomyelin concentrations seen in both cohorts might be relevant for cardiovascular risk assessment, given the established links between elevated sphingomyelins and subclinical atherosclerosis and coronary artery disease.

## Data Availability

The data generated and analyzed in the presented study are available from the corresponding author on request.
